# Assessing Emotion Regulation Strategies in Chile: A Spanish Language Adaptation of the German SSKJ 3-8 Scales

**DOI:** 10.3389/fpsyg.2019.02870

**Published:** 2020-01-09

**Authors:** Mirjam Weis, Jörg-Henrik Heine

**Affiliations:** TUM School of Education, Centre for International Student Assessment (ZIB), Technical University of Munich, Munich, Germany

**Keywords:** emotion regulation, Chile, measuring invariance, item response theory, cross-cultural research

## Abstract

The aim of the present study is the psychometric review of a Spanish language version of the German Stress and Coping Questionnaire for Children and Adolescents (SSKJ 3-8). The SSKJ 3-8 assesses emotion regulation strategies by asking children to indicate how often they use five different emotion regulation strategies in response to a social and an academic stress situation. The Spanish language adaptation of the inventory was developed for a cross-cultural study with Chilean and German fourth-graders. The sample includes 76 German and 169 Chilean fourth graders. The SSKJ 3-8 was first translated into Spanish by a bilingual translator, then back-translated by a German native speaker, and finally adapted for the cultural context in Chile. To analyze the psychometric comparability, the measurement invariance was examined within the framework of the Item Response Theory (IRT) with the R package pairwise. The results show that the new developed Spanish language adaptation of the SSKJ 3-8 is comparable to the German version in terms of psychometric measurement characteristics. Only few items show deviations with regard to strong measurement invariance. We conclude that the Spanish language adaptation of the SSKJ 3-8 is a reliable instrument to assess emotion regulation strategies in Chile.

## Introduction

Children’s emotion regulation is critical for children’s socioemotional and cognitive functioning as well as an important indicator for positive developmental outcomes (Calkins, 2007). Emotion regulation has been defined as a process through which emotions are managed (i.e., initiated, inhibited, avoided, maintained, or modulated) to achieve biological or social adaptations, or individual goals (Eisenberg and Spinrad, 2004). Emotion regulation can be understood as process (“*emotion as regulating”*) through which emotions lead to changes as well as in terms of strategies (“*emotion as regulated”*) that aim to change the experience of emotions (Cole et al., 2004). The “Stress and Coping Questionnaire for Children and Adolescents” (SSKJ 3-8, Lohaus et al., 2006) refers on the transactional model of Lazarus and Folkman (1984) and includes problem- as well as emotion-oriented strategies. While emotion-oriented strategies intend to reduce the experience of negative emotions, problem-oriented strategies intend to change a specific situation, which evoked the negative emotions (Lohaus et al., 2006). The SSKJ 3-8 assesses strategies of emotion regulation for coping with negative emotions in third to eight graders. These are called “coping strategies” in the original manual (Lohaus et al., 2006; Eschenbeck et al., 2012). We refer to them as emotion regulation strategies, understanding them as strategies, which intend to change the experience of negative emotions (“*emotion as regulated”*; Cole et al., 2004), distinguishing between problem-oriented and emotion-oriented strategies to manage negative emotions (Lazarus and Folkman, 1984). In addition, the SSKJ 3-8 contains scales on stress vulnerability and stress symptoms (Lohaus et al., 2006; Eschenbeck et al., 2012), which we did not consider in the present study.

The questionnaire comprises the following five emotion regulation strategies: *Avoidant coping (VER)*, *palliative strategies (KON)*, *anger-oriented strategies (DES)*, *seeking social support (SOZ)*, and *problem solving (PRO)* (Lohaus et al., 2006; Eschenbeck et al., 2012).*VER*, *KON*, and *DES* are included as emotion-oriented strategies, which are understood as directly changing the experience of negative emotions. While the focus of *KON* is on relaxation and distraction, *DES* mean externalizing emotions of anger and rage. As problem-oriented strategies are directed to the context and are defined as instrumental actions to change the cause of the negative emotion, *SOZ* and *PRO* are included (Lohaus et al., 2006; Eschenbeck et al., 2012).

The assumption is, that none of the specific strategies is *per se* superior or inferior, but rather that the adequacy of emotion regulation strategies depends on the situation (Lazarus and Folkman, 1984; Lohaus et al., 2006). Thus, assessments of emotion regulation strategies should include several situations in order to test the capacity of using specific strategies in a flexible way, depending on the situation (Causey and Dubow, 1992; Lohaus et al., 2006). Therefore, the SSKJ 3-8 differentiates between situations and includes a social as well as an academic situation (Lohaus et al., 2006). However, the test manual of the SSKJ 3-8 (Lohaus et al., 2006) proposes that a summative evaluation across the two situations as well as a situation specific evaluation of the SSKJ 3-8 are possible. The factor structure of the German Version of the SSKJ 3-8, including the mentioned five different emotion regulation strategies as well as the two stressful situations, which elicit negative emotions, has been confirmed in a study with 1991 third to eight graders. Further, the German SSKJ 3-8 has been shown to be a reliable and valid instrument (Eschenbeck et al., 2006). The questionnaire is useful for prevention as well as intervention purposes in all areas in which children or adolescents experience increased levels of stress (Lohaus et al., 2006). For instance, when there are already indications for problems in a child’s emotion regulation, the SSKJ 3-8 questionnaire can help to specify these and point out to supportive intervention methods. Thus, this questionnaire is relevant for research and practice in developmental, clinical, and health psychology (Lohaus et al., 2006).

Emotion regulation strategies might differ between cultural contexts because of culture specific socialization practices (Trommsdorff, 2009; Jaramillo et al., 2017). Thus, it is important to consider aspects of the cultural context, when applying instruments to measure emotion regulation strategies. In the present study, the SSKJ 3-8 was applied in Germany and Chile to assess fourth graders emotion regulation strategies. The cultural context of Germany is typically defined as an independent sociocultural context, in which interdependent values are prioritized and people strive for individual autonomy (Hofstede, 2001; Trommsdorff, 2009). According to Hofstede (1980, 2001), Germany can be ranked as a country with high individualist values. For Chile, the classification is less clear. Although, Chile has been ranked as one of the most collectivistic countries by Hofstede (1980), more recent studies showed that in Chile both independence and interdependence are valued highly (e.g., Kolstad and Horpestad, 2009) and influence parenting and child development (Bush and Peterson, 2014). The coexistence of independence and interdependence might be rooted in political, economic, and societal changes of the last decades in Chile (Martínez et al., 2006). According to cultural psychological theories (e.g., Kağitçibasi, 1996; Trommsdorff and Kornadt, 2003), economic and societal changes might lead to a combination and multidimensionality of independent and interdependent values, for instance in contexts in which material dependence decreases while emotional relatedness remains important. Further, the Latin-American values *simpatía* (to respect and share other’s feelings), *familismo* (family commitment, strong family bonds), and *respeto* (to avoid negative behaviors) might motivate Chileans for interpersonal harmony (Triandis et al., 1984; Halgunseth et al., 2006).

Regarding specific emotion regulation strategies like DES have been shown to differ among cultural contexts (Trommsdorff and Cole, 2011). For instance, in a study with German and Chilean fourth graders, German children reported to use DES more often than Chilean children (Weis et al., 2016). While in interdependent contexts as well as based on Latin-American values (simpatía, respeto) interpersonal harmony is encouraged and negative emotional expressions as anger are avoided, in independent contexts the expression of anger might be instrumental to achieve individual goals (Triandis et al., 1984; Halgunseth et al., 2006; Trommsdorff, 2009). Differences in anger-oriented emotion regulation strategies between cultural groups might stem from differences in parenting practices. Cole et al. (2006) found in a study with child-adults dyads of two different cultural groups in Nepal, differences in caregivers responses (rebuking vs. reasoning and yielding) to children’s anger expressions. In general, in independent context, for instance in Germany, children are encouraged by parents to express emotions as dissatisfaction or anger in order to contribute to children’s development of self-assertiveness. In interdependent context, for instance in Asian countries, parents undermine reactions of negative emotions by downplaying the frustration event and helping to accept the situation (Trommsdorff, 2009; Jaramillo et al., 2017). Parenting practices are guided by intuitive theories on socioemotional processes, which are influenced by cultural values (Trommsdorff, 2009; Trommsdorff and Cole, 2011). These culturally influenced parenting practices in turn could affect children’s appraisal of emotional experiences as well as the selection and application of specific emotion regulation strategies (Diaz and Eisenberg, 2015). However, children develop emotion regulation not only through socialization in the family, but also in the school context, through cultural products (e.g., books and other media), and language acquisition (Savina and Wan, 2017).

As emotion regulation strategies might differ across cultural contexts, it is important to use cultural adequate instruments for diagnostic purposes as well as for research. For cross-cultural studies on emotion regulation strategies, it is essential to assure equivalence of instruments in order to ensure comparability of data of multiple cultural contexts (van de Vijver and Leung, 1997; He and van de Vijver, 2012). To our knowledge, there exist few scales on emotion regulation strategies of children and adolescents in several languages with psychometric evidence for cultural equivalence. The present research aims to evaluate the psychometric quality and equivalence of a Spanish language adaptation of the emotion regulation strategies from the German SSKJ 3-8 (Lohaus et al., 2006) in comparison with the original German version. Therefore, we applied the SSKJ 3-8 in Germany and Chile to assess fourth graders’ emotion regulation strategies. The development of the Spanish version included a translation into Spanish language as well as a careful cultural adaptation of the questionnaire considering the cultural context of the target population in Chile. The final aim was to contribute a Spanish version as a reliable instrument to assess emotion regulation strategies, which might be useful for diagnostic purposes of practitioners as well as for developmental, clinical, and cross-cultural psychological research.

A previous study validated a Turkish adaptation of the German SSKJ 3-8 in a sample of children and adolescents in Turkey (Eschenbeck et al., 2012). The findings of the study in Turkey indicate that the SSKJ 3-8 seems to work in other cultural contexts, besides Germany. While Eschenbeck et al. (2012) focused on the confirmation of the factor structure, correlations with indicators of psychological adjustment, and replication of gender differences, the present study focuses on scalability and measurement invariance to analyze the psychometric quality of the Spanish adaptation.

As past research emphasized to assess emotion regulation strategies separately for specific situations (Causey and Dubow, 1992; Lohaus et al., 2006), we hypothesized that distinguishing two different situations (i.e., social and academic situation) for the five scales of the SSKJ 3-8 holds across cultural contexts (i.e., Chilean and German subsamples). Thus, we expected that a scaling model divided by the two situations fits better than a scaling model across the two situations (hypothesis 1). Second, we hypothesized that due to the careful translation and cultural adaptation, there will be little differential item functioning (DIF) between the new Spanish adaptation and the German original version of the SSKJ 3-8 (hypothesis 2) as well as satisfactory reliabilities based on the total sample of the German and Chilean subsamples. Moreover, we discuss if there are differences between the Chilean and German subsamples in particular item characteristics because of cultural differences.

## Materials and Methods

### Participants and Procedure

In total, 245 fourth graders participated in the study. The sample in Germany included 76 children (31 boys, 45 girls) and the sample in Chile included 169 children (56 boys, 113 girls). Children’s mean age was 10.21 years (*SD* = 0.44) in Germany and 10.16 years (*SD* = 0.42) in Chile. For Germany, we drew the sample from seven different fourth grade classes, in four primary schools in a medium-sized town in Southern Germany. In Chile, we conducted the study in nine different fourth grade classes, in four primary schools (two public, two private) in a large city in Central Chile. 243 mothers of the children (76 in Germany, 167 in Chile) responded to questions on demographic information. Mothers’ level of education was categorized according to the International Standard Classification of Education (ISCED-97; OECD, 1999). In Germany, 6.6% of the mothers had completed lower secondary level of education (=2), 13.2% upper secondary level (=3), 30.3% post-secondary (=4), and 50% had completed first stage of tertiary education (=5). In the sample in Chile, 1.8% of the mothers had completed no school leaving certificate (=0), 10.2% primary level of education (=1), 29.3% lower secondary level of education (=2), 28.7% upper secondary level of education (=3), and 29.9% had completed first stage of tertiary education (=5). Thus, with regard to our sample, the mothers’ educational level was lower for mothers in Chile than in Germany, which corresponds to population differences in socioeconomic status of families between the countries (e.g., OECD, 2016, p. 401).

### Assessment of Emotion Regulation Strategies With the SSKJ 3-8

The SSKJ 3-8 is a stimulus-response self-report questionnaire, in which children are asked to imagine being in a social and in an academic stressful situation, which evoke negative emotions. The social stressful situation is about arguing with a friend (situation A; Spanish translation: Imagínate que tuviste una gran pelea con un buen amigo o una buena amiga.) and the academic situation is problems with homework (situation B; Spanish translation: Imagínate que tienes muchísimas tareas y que no sabes qué hacer para terminarlas.) (Lohaus et al., 2006; Eschenbeck et al., 2012). Children indicate on a five-point rating scale how often (from 1 = *never* to 5 = *always*) they use five different emotion regulation strategies in response to these two situations to regulate their emotions (Lohaus et al., 2006; Eschenbeck et al., 2012). The SSKJ 3-8 includes the five emotion regulation strategies *seeking social support* (Suche nach sozialer Unterstützung, SOZ; e.g., “I ask someone for help”^1^), *problem solving* (Problemorientierte Bewältigung, PRO; e.g., “I try to think of different ways to solve it”), *avoidant coping* (Vermeidende Bewältigung, VER; e.g., “I tell myself it doesn’t matter”), *palliative strategies* (Konstruktiv-palliative Strategien, KON; e.g., “I take a rest”), and *anger-oriented strategies* (Destruktiv-ärgerbezogene Strategien, DES; e.g., “I get mad and break something”; Lohaus et al., 2006; Eschenbeck et al., 2012). Each emotion regulation strategy is operationalized by six items per situation (A and B). Thus, 12 items for each of the five emotion regulation strategies across the two situations and consequently 60 items in total are included (Lohaus et al., 2006; Eschenbeck et al., 2012). The factor structure of the German version of the SSKJ 3-8 has been confirmed and sufficient reliability as well as validity has been proved (e.g., Eschenbeck et al., 2006).

### Translation and Cultural Adaptation

We developed the Spanish adaptation of the German SSKJ 3-8 inventory in a cross-cultural study with fourth-graders in Chile and Germany (Weis et al., 2016). In a first stage, a bilingual German-Chilean with German and Spanish as native languages translated the items of the five strategies of emotion regulation as well as the two situations of the German source version of the SSKJ 3-8 by Lohaus et al. (2006) into Spanish. Next, a German native speaker back translated the Spanish version into German. After the back translation, we discussed all discrepancies, which emerged, until agreement was reached.

In an additional step, we checked the translated Spanish language version with regard to cultural aspects of the specific target population and carefully adapted the respective items accordingly. Local Chilean psychologists and teachers, who were native speakers, revised the Spanish language version of the questionnaire. The first author discussed the content of the items and language characteristics with the native teachers and psychologists until the discussion reached agreement. For instance, labels for answer categories of the rating scale from 1 = *never* to 5 = *always* were given for each item instead of giving the five labels only on top of each page as in the German version.

Next, to test and ensure the cultural adaptation of the developed Spanish version of the questionnaire, we conducted a pilot study with 12 fourth graders (5 female, 7 male) in Chile. In this pilot study, we evaluated the wording and understandability of the items. The pilot study showed that the fourth graders in Chile generally understood the questionnaire. However, there were two items (“I curse to myself.”; “I allow myself to take a break.”),^2^ which were not understood by some participants. Thus, we again discussed these two items with local psychologists and changed the item wording to be more comprehensible for the target population.

Finally, to ensure culturally appropriateness of the testing procedure and to avoid administration bias (He and van de Vijver, 2012), the questionnaire was administered in the schools by local psychologists and psychology students of the research team in Chile. Herewith, we assured cultural appropriate instructions as well as cultural adequate interactions between administrator and respondents. Further, the first author, who was part of the administration team in the German study, was present at the administration in Chile to ensure comparability of all administration conditions.

### Data Analysis

To analyze the psychometric quality of the Spanish adaptation of the SSKJ 3-8, we examined the measurement invariance in the context of Item Response Theory (IRT). The IRT framework offers the possibility to test for the highest level of measurement equivalence, which is according to van de Vijver and Tanzer (2004) termed as *scalar* equivalence or *full scale* equivalence. This level of equivalence includes structural equivalence, which is in the area of cross-cultural psychology according to Triandis and Marín (1983) in turn associated with an “etic” position. Against the backdrop of the rather small sample sizes available in these analyses, we choose the package *pairwise* (Heine, 2019) for the open source statistical environment *R* (R Core Team, 2019) for all IRT scaling tasks. The package *pairwise* offers a non-iterative method for item parameter calibration named *PAIR* (see e.g., Choppin, 1968; Fischer, 1970; Fischer and Scheiblechner, 1970a; Wright and Masters, 1982), which according to Heine and Tarnai (2015) returns stable item parameter estimates even based on rather small sample sizes (see also Heine et al., 2018 for an practical application). Subsequent to the process of item calibration, person parameters estimation can base on the weighted likelihood approach introduced by Warm (1989).

In order to test hypothesis 1, we applied several one-dimensional scaling approaches based on the Partial-Credit-Model (PCM – Masters, 1982) for polytomous item answer scales. We analyzed each of the five scales based on the PCM (Masters, 1982), applying a one-dimensional scaling approach. On the one hand, the items of the five scales (i.e., five emotion-regulation strategies) were included across both stressful situations (i.e., academic and social situation). Thus, 12 items were included for each scale. On the other hand, we divided the items sets according to the two situations (i.e., six items for each of the five scales per situation) and analyzed the resulting scales separately in a one-dimensional scaling approach.

In the first scaling approach, the global model fit was evaluated by applying the Andersen Likelihood Ratio Test (Andersen, 1973) on the 12 items for each of the five scales across both stressful situations, using the splitting criterion of the cultural context (i.e., Germany – Chile). Additionally, we report the residual based model fit statistics *Q3* (see Yen, 1984), based on the separate scaling approaches for each of the five scales and situations, respectively. To analyze any local model violations on item level, we calculated root-mean-square statistics (INFIT and OUTFIT – e.g., Wright and Masters, 1982). Based on the fixed item parameter estimates resulting from the concurrent calibration approach across both cultural contexts (i.e., based on the total sample), the INFIT and OUTFIT statistics were calculated and evaluated separately for each subsample (i.e., for each cultural context). To detect any sub-dimensionality of the five scales, possibly resulting from the two stressful situations (social situation vs. academic situation), we performed a Rasch-Residual-Factor-Analysis (RRFA – Wright, 1996; Linacre, 1998). Therefore, we calculated the Rasch-Residual matrices, resulting from the one-dimensional scaling across the 12 items for each of the five scales and analyzed them applying a principal component analysis (Wright, 1996). We plot the item difficulty against the loadings on the first main component to examine if there is any clear assignment of the respective item residuals to the two stressful situations.

In the second scaling approach, the items were scaled separately for the two situations (social situation vs. academic situation, A vs. B) and five dimensions. Thus, we applied a one-dimensional scaling model (PCM) for each of the ten resulting scales. For each of the ten scales (six items per scale), we computed the residual based model fit statistics *Q3* (see Yen, 1984) to evaluate the global model fit. To detect any local model deviations we computed INFIT and OUTFIT statistics for each of the ten scales.

To examine the measurement invariance across both cultural contexts, the Fischer-Scheiblechner *Z*-test (Fischer and Scheiblechner, 1970b; van den Wollenberg, 1982) was performed for all ten scales to test for DIF between sub-samples (Germany – Chile).

## Results

### Scaling Models for the SSKJ

Overall, the results indicate that the five scales across the two situations of SSKJ 3-8 show satisfactory reliabilities based on the total sample (Germany and Chile). The reliabilities of the weighted likelihood estimates (WLE; Warm, 1989; Andrich, 1988) for the five scales range from *r*_WLE_ = 0.79 (scale DES) to *r*_WLE_ = 0.86 (scale KON). However, the Andersen test turned out to be significant for every scale except for SOZ, suggesting an overall misfit of the scaling model across the two situations for the four other scales (see Table 1). In line with the findings from the Andersen test, the coefficients for the Yen‘s Q3 statistics turned out to fall below the recommended limit of *r*_Q3 – max_ < 0.2 (see Yen, 1984) only for the scale SOZ (SOZ: *r*_Q__3–__max_ = 0.194, PRO: *r*_Q__3–__max_ = 0.369, KON: *r*_Q__3–__max_ = 0.272, VER: *r*_Q__3–__max_ = 0.209, DES: *r*_Q__3–__max_ = 0.398).

**TABLE 1:** Coefficients for local and global fit indices for one dimensional scaling across both situations for five scales.

	**Germany**					**Chile**					***Andersen Global Fit***			***Reliability***
**Scale/Item**	**χ^2^**	***df***	***p*_χ__2_**	***OUTFIT*_zSTD_**	***INFIT*_zSTD_**	**χ^2^**	***df***	***p*_χ__2_**	***OUTFIT*_zSTD_**	***INFIT*_zSTD_**	**χ^2^**	***df***	***p*_χ__2_ _And._**	***r*_WLE_**
DES A1	64.23	73	0.76	0.26	0.34	164.91	168	0.55	0.39	0.33	244.91	95	0.00	0.79
DES A2	63.80	74	0.80	0.20	0.44	138.51	168	0.95	−0.39	−0.73				
DES A6	57.17	74	0.93	−0.28	−0.51	174.94	167	0.32	1.07	0.66				
DES A3	67.11	74	0.70	0.36	0.67	154.30	168	0.77	0.05	0.43				
DES A4	75.08	73	0.41	1.03	1.92	194.63	167	0.07	1.87	0.88				
DES A5	35.40	75	1.00	−0.66	−1.13	126.51	167	0.99	−0.44	0.28				
DES B1	50.53	75	0.99	−0.42	−0.11	136.36	168	0.96	−0.48	−0.37				
DES B2	102.49	74	0.02	1.48	0.77	125.33	168	0.99	−0.77	−1.09				
DES B6	57.80	74	0.92	−0.15	0.20	109.95	167	1.00	−1.89	−2.31				
DES B3	70.90	74	0.58	0.48	0.48	127.56	167	0.99	−0.74	−0.27				
**DES B4**	46.59	74	0.99	−0.91	−0.75	283.73	167	**0.00**	**4.50**	**2.97**				
DES B5	48.49	75	0.99	−0.23	−1.05	112.05	167	1.00	−0.75	0.07				

KON A1	57.42	74	0.92	−1.05	−0.54	180.53	168	0.24	0.26	−0.34	214.88	95	0.00	0.86
KON A2	74.39	74	0.47	0.40	0.39	189.48	168	0.12	0.66	0.68				
**KON A3**	143.60	72	**0.00**	4.40	**3.73**	231.61	168	**0.00**	**2.70**	**2.50**				
KON A4	72.89	74	0.51	0.28	0.73	155.40	168	0.75	−1.12	−0.74				
KON A6	72.88	73	0.48	0.38	0.52	173.84	168	0.36	−0.08	−0.45				
KON A5	54.19	73	0.95	−1.37	−0.97	157.90	167	0.68	−0.94	−0.73				
KON B1	54.14	74	0.96	−1.26	−0.99	150.16	168	0.83	−1.37	−1.35				
KON B2	61.75	74	0.84	−0.65	−1.51	190.29	168	0.11	0.72	1.32				
KON B3	61.94	74	0.84	−0.65	−0.53	194.00	168	0.08	0.96	0.60				
**KON B4**	73.05	73	0.48	0.36	0.88	129.95	168	0.99	−**2.24**	−**2.51**				
KON B6	49.02	75	0.99	−1.96	−1.93	191.08	168	0.11	0.74	1.55				
KON B5	58.33	74	0.91	−1.02	−0.73	168.92	166	0.42	−0.25	−0.76				

**PRO A1**	43.76	75	1.00	−0.55	−0.83	259.53	167	0.00	**2.49**	1.80	181.40	95	0.00	0.85
PRO A2	48.58	73	0.99	−0.11	−0.10	222.70	168	**0.00**	1.66	1.43				
PRO A3	42.78	73	1.00	−0.57	−0.72	172.44	168	0.39	−0.52	−0.96				
**PRO A4**	55.41	75	0.96	0.32	0.43	129.71	167	0.99	−**2.25**	−**2.10**				
PRO A5	44.30	74	1.00	−0.40	−0.41	153.16	168	0.79	−1.13	−0.59				
PRO A6	43.87	73	1.00	−0.49	−0.62	197.68	168	0.06	0.55	−0.12				
**PRO B1**	90.59	75	0.11	**2.32**	**2.26**	172.40	166	0.35	−0.36	−0.21				
PRO B2	42.82	75	1.00	−0.66	−0.59	167.09	168	0.51	−0.73	−0.42				
PRO B3	62.49	75	0.85	0.78	0.81	148.47	168	0.86	−1.36	−1.09				
PRO B4	37.55	74	1.00	−0.93	−0.74	151.91	167	0.79	−1.22	−0.62				
**PRO B5**	51.09	74	0.98	0.06	0.31	218.41	167	**0.00**	1.16	1.85				
**PRO B6**	51.90	74	0.98	0.12	−0.06	224.55	168	**0.00**	1.51	1.08				

VER A1	52.35	74	0.97	−0.06	0.07	204.75	168	0.03	1.64	0.37	182.74	95	0.00	0.79
VER A2	58.91	74	0.90	0.39	0.66	152.28	167	0.79	−1.07	−1.12				
VER A3	59.64	74	0.89	0.37	0.64	200.29	168	0.05	1.26	1.79				
**VER A4**	49.79	75	0.99	−0.14	0.33	119.72	168	1.00	−**2.13**	−**2.12**				
VER A5	62.40	73	0.81	0.69	0.61	173.25	166	0.33	0.20	0.31				
VER A6	76.47	75	0.43	1.30	1.16	165.34	168	0.54	−0.33	0.32				
VER B1	49.72	75	0.99	−0.27	−0.21	178.60	167	0.26	0.40	−0.05				
VER B2	50.43	74	0.98	−0.18	−0.43	206.42	168	0.02	1.67	0.18				
VER B3	49.85	75	0.99	−0.15	−0.08	145.64	168	0.89	−1.07	−0.33				
VER B4	36.76	74	1.00	−0.51	−0.69	118.33	167	1.00	−1.56	−0.62				
VER B5	51.90	74	0.98	−0.12	−0.24	199.19	168	0.05	1.51	1.44				
VER B6	55.82	75	0.95	0.13	−0.74	199.88	168	0.05	1.16	0.74				

SOZ A1	62.21	74	0.83	0.45	0.37	212.49	168	0.01	1.51	1.38	98.58	95	0.38	0.85
SOZ A2	47.05	74	0.99	−0.68	−0.96	175.05	168	0.34	−0.12	−0.29				
SOZ A3	54.08	74	0.96	−0.11	−0.12	173.29	167	0.35	−0.15	0.13				
SOZ A4	50.90	75	0.99	−0.42	−0.78	153.28	168	0.79	−1.28	−1.28				
SOZ A5	49.98	75	0.99	−0.52	−0.47	160.72	167	0.62	−0.87	−1.02				
SOZ A6	50.80	74	0.98	−0.37	−0.53	160.42	168	0.65	−0.89	−1.00				
**SOZ B1**	81.29	75	0.29	1.38	1.79	249.09	168	**0.00**	**2.43**	**2.78**				
SOZ B2	67.19	74	0.70	0.81	0.61	163.48	168	0.58	−0.74	−0.46				
SOZ B3	56.44	74	0.94	0.08	0.52	188.73	168	0.13	0.50	0.59				
SOZ B4	55.03	73	0.94	0.01	0.13	144.65	168	0.90	−1.77	−1.41				
SOZ B5	57.91	74	0.92	0.17	0.25	176.10	166	0.28	0.05	0.23				
SOZ B6	46.93	75	1.00	−0.64	−0.70	174.67	168	0.35	−0.12	−0.33				

**n* = 245 (Chile: *n* = 169; Germany: *n* = 76); DES = anger-oriented strategies; KON = palliative strategies; PRO = problem solving; SOZ = seeking social support; VER = avoidant coping; Situation A = social situation (arguing with a friend); Situation B = academic situation (problems with homework); *p*_χ__2_ = *p*-value for pearson χ^2^ test; *OUTFIT*_*MSQ*_ = outfit-mean-square statistic; *INFIT*_*MSQ*_ = infit-mean-square statistic; *OUTFIT*_*zSTD*_ = *Z*-standardized outfit statistic; *INFIT*_*zSTD*_ = *Z*-standardized infit statistic, values above 1.964 or below −1.964 in bold face; concurent calibration approach for itemparameter estimation based on the total sample (Germany and Chile); *r*_*WLE*_ = reliabilities of the weighted likelihood estimates.*

**FIGURE 1:**
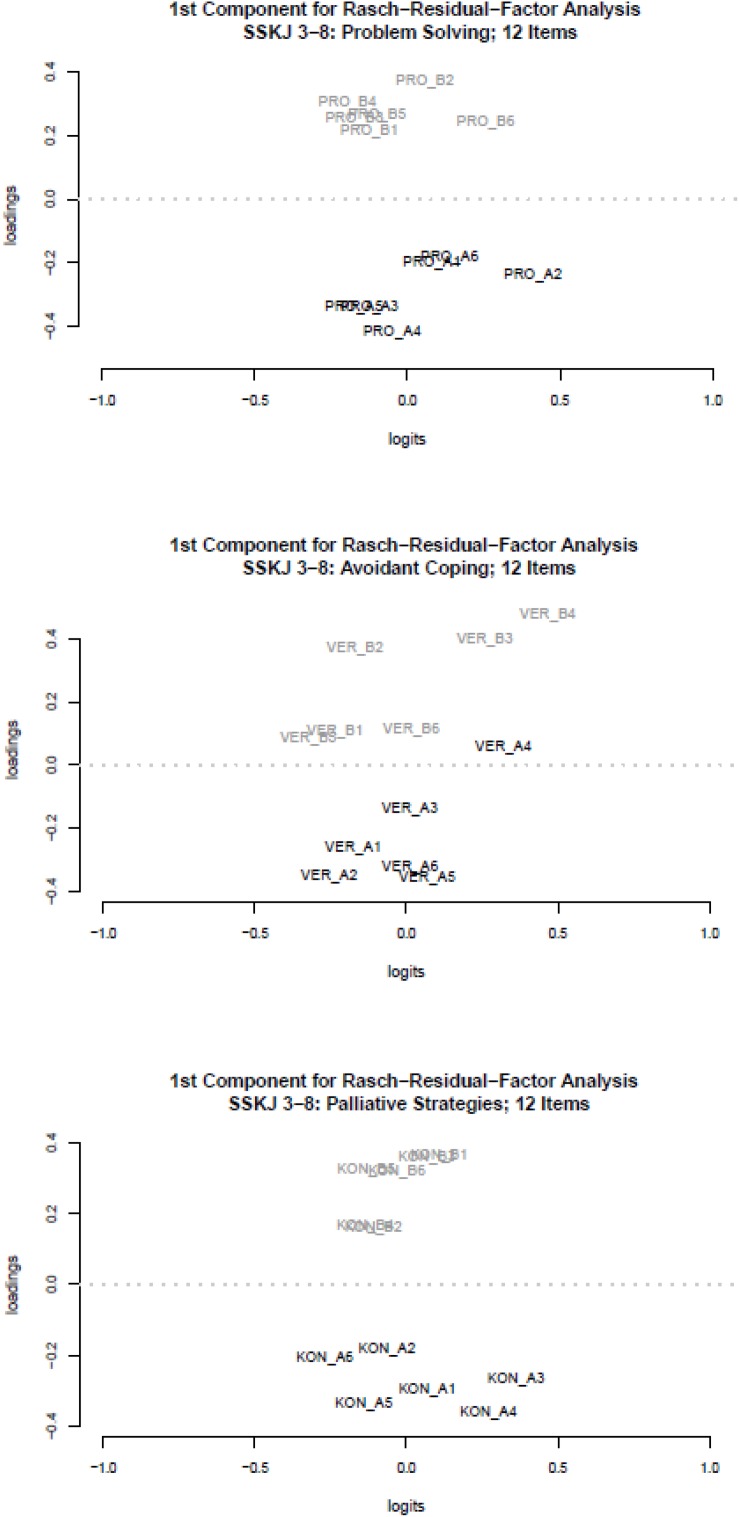
Rasch-Residual-Factor Analysis: Loadings on the first component (*y*-axis) and item difficulties in Logits (*x*-axis) for scale PRO **(top panel)**, scale VER **(middle panel)** and scale KON **(bottom panel)**; A = social situation (arguing with a friend), B = academic situation (problems with homework).

Contrary to the findings of the global model fit, local model fit tested by root-mean-square fit-statistics and χ^2^-tests across both subsamples and across the five scales, identified only ten of the 60 items, which showed significant deviations from the underlying scaling model (see Table 1). The loading pattern out of RRFA (Wright, 1996; Linacre, 1998) indicates a clear assignment of the items to one of the two situations (social situation vs. academic situation) for three of the five scales (see Figure 1).

**FIGURE 2:**
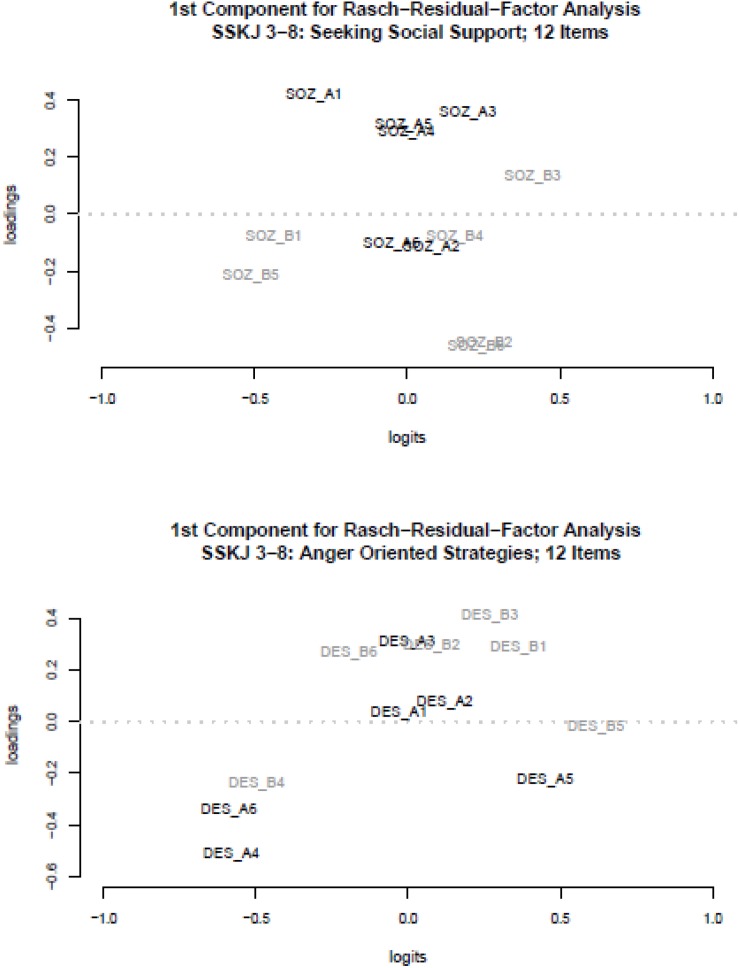
Rasch-Residual-Factor Analysis: Loadings on the first component (*y*-axis) and item difficulties in Logits (*x*-axis) for scale SOZ **(top panel)** and scale DES **(bottom panel)**; A = social situation (arguing with a friend), B = academic situation (problems with homework).

These loading patterns on the first main component of the RRFA indicate a substantive sub-dimensionality of the three scales, which is associated to the two stressful situations. In contrast to the findings for the three subscales depicted in Figure 1, a different picture emerged for the two scales SOZ and DES. For both, the RRFA resulted in a rather undifferentiated loading pattern with regard to the two stressful situations (see Figure 2).

Specifically, the ambiguous loading patterns for SOZ and DES let not suggest a clear and evident interpretation with regard to a substantive sub-dimensionality related to the two stressful situations. While the scales PRO and KON showed for both countries (see Table 1) a deviation of the unidimensional scaling model across both situations, the scale SOZ seems to match the model assumption quite good (no significant Andersen test, Yen’s fit statistic Q3 was *r*_Q__3–__max_ < 0.2). However, the scale SOZ shows some minor model deviations with regard to item SOZ B1 for the Chilean subsample. Consequently, we analyzed the items divided by the two situations (social vs. academic situation, A vs. B).

For the subsequent scaling approach, which differentiates between the two situations, the overall global model fit across both countries, as indicated by Yen‘s Q3 statistics fell below the recommended limit of *r*_Q__3–__max_ < 0.2 (see Yen, 1984) for all of the ten scales (see Table 2). Contrary, the global model fit according to Yen’s Q3 based on the separated two subsamples (Germany, Chile) fell beyond the limit of *r*_Q__3–__max_ < 0.2 for only four scales within the German subsample (DES A *r*_Q__3–__max_ = 0.213; KON B *r*_Q__3–__max_ = 0.243; SOZ A *r*_Q__3–__max_ = 0.262; SOZ B *r*_Q__3–__max_ = 0.222) as depicted in Table 2.

**TABLE 2:** Coefficients for Q3 statistic for the total sample by country and subscale.

	**DES**		**KON**		**PRO**		**SOZ**		**VER**	
	**A**	**B**	**A**	**B**	**A**	**B**	**A**	**B**	**A**	**B**
Total sample	0.02	0.09	0.05	0.07	0.10	0.07	−0.01	0.04	−0.06	0.07
Chile	0.03	0.13	−0.01	0.06	0.13	0.06	−0.04	0.01	−0.07	0.05
Germany	**0.21**	0.10	0.13	**0.24**	0.13	0.09	**0.26**	**0.22**	0.08	0.10

**n* = 245 (Chile: *n* = 169; Germany: *n* = 76); DES = anger-oriented strategies; KON = palliative strategies; PRO = problem solving; SOZ = seeking social support; VER = avoidant coping; Situation A = social situation (arguing with a friend); Situation B = academic situation (problems with homework); coefficients > 0.2 in bold face.*

### Measurement Invariance and Differences Between Chilean and German Subsamples

Regarding measurement invariance, for the most items no DIF was found. As can be seen in Table 3, for only ten of the 60 items, the Fischer-Scheiblechner *Z*-test turned out to be significant (DES: A4; B4; KON: B1, B3, B4, A3, A6; VER: B3, B4; SOC: B4) when splitting the sample by country. Moreover, based on the root mean square statistic only five items showed significant deviations (SOZ: B1; PRO: A1; KON: A3; DES: A4, B4).

**TABLE 3:** Coefficients for Fisher-Scheiblechner *Z*-Test, local and global fit indices for one dimensional scaling differenciated by situations.

								***Fisher-Scheiblechner***		***Reliability***
**Scale/Item**	**χ^2^**	***df***	***p*_χ__2_**	***OUTFIT*_MSQ_**	***OUTFIT*_zSTD_**	***INFIT*_MSQ_**	***INFIT*_zSTD_**	***Z***	***p***	***r*_WLE_**
SOZ A1	274.71	243	0.08	1.17	1.48	1.10	1.32	1.33	0.18	0.79
SOZ A2	222.10	243	0.83	0.95	−0.51	0.94	−0.78	−0.87	0.39	
SOZ A3	227.37	242	0.74	0.98	−0.22	1.00	0.04	0.93	0.35	
SOZ A4	204.19	244	0.97	0.87	−1.33	0.89	−1.53	−1.05	0.29	
SOZ A5	210.70	243	0.93	0.90	−1.05	0.91	−1.13	−1.06	0.29	
SOZ A6	211.22	243	0.93	0.91	−0.97	0.91	−1.16	0.16	0.87	

SOZ B1	330.37	244	0.00	1.39	**2.76**	1.30	**3.22**	0.60	0.55	0.76
SOZ B2	230.67	243	0.70	0.98	−0.13	1.00	−0.04	0.73	0.47	
SOZ B3	245.17	243	0.45	1.04	0.41	1.07	0.82	0.29	0.77	
**SOZ B4**	199.68	242	0.98	0.86	−1.42	0.92	−1.08	−2.68	**0.01**	
SOZ B5	234.01	241	0.61	1.01	0.10	1.02	0.25	0.82	0.41	
SOZ B6	221.60	244	0.85	0.94	−0.51	0.95	−0.65	−1.73	0.08	

PRO A1	303.29	243	0.01	1.27	**2.06**	1.08	0.97	0.62	0.53	0.81
PRO A2	271.28	242	0.10	1.15	1.43	1.10	1.17	−0.51	0.61	
PRO A3	215.22	242	0.89	0.92	−0.74	0.90	−1.18	−0.02	0.98	
PRO A4	185.11	243	1.00	0.79	−1.80	0.88	−1.38	0.10	0.92	
PRO A5	197.46	243	0.99	0.84	−1.21	0.93	−0.76	0.37	0.71	
PRO A6	241.55	242	0.50	1.03	0.26	0.96	−0.46	−0.64	0.53	

PRO B1	262.99	242	0.17	1.11	0.91	1.13	1.31	−1.23	0.22	0.78
PRO B2	209.91	244	0.94	0.89	−0.99	0.94	−0.73	0.58	0.56	
PRO B3	210.96	244	0.94	0.89	−0.82	0.96	−0.38	−0.13	0.89	
PRO B4	189.46	242	0.99	0.81	−1.55	0.91	−0.96	0.31	0.75	
PRO B5	269.49	242	0.11	1.14	1.05	1.15	1.64	0.24	0.81	
PRO B6	276.45	243	0.07	1.16	1.39	1.07	0.79	0.05	0.96	

KON A1	237.94	243	0.58	0.97	−0.34	0.95	−0.59	0.19	0.85	0.79
KON A2	263.87	243	0.17	1.07	0.75	1.06	0.74	−0.11	0.91	
**KON A3**	375.21	241	0.00	1.54	**4.90**	1.37	**4.21**	5.02	**0.00**	
KON A4	228.28	243	0.74	0.93	−0.77	0.98	−0.18	−1.57	0.12	
KON A5	212.08	241	0.91	0.87	−1.53	0.91	−1.18	−1.73	0.08	
**KON A6**	246.72	242	0.40	1.01	0.10	0.99	−0.10	−2.16	**0.03**	

**KON B1**	204.30	243	0.97	0.83	−1.86	0.88	−1.66	2.38	**0.02**	0.81
KON B2	252.04	243	0.33	1.02	0.28	1.02	0.31	1.01	0.31	
**KON B3**	255.94	243	0.27	1.04	0.47	1.02	0.22	2.81	**0.00**	
**KON B4**	203.00	242	0.97	0.83	−1.69	0.88	−1.51	−2.42	**0.02**	
KON B5	227.25	241	0.73	0.93	−0.76	0.92	−1.06	−0.53	0.60	
KON B6	240.11	244	0.56	0.97	−0.27	1.03	0.35	−0.43	0.67	

VER A1	257.10	243	0.26	1.13	1.23	1.02	0.29	−1.12	0.26	0.70
VER A2	211.19	242	0.92	0.94	−0.62	0.95	−0.64	−0.79	0.43	
VER A3	259.93	243	0.22	1.14	1.16	1.16	1.84	0.21	0.83	
VER A4	169.51	244	1.00	0.76	−1.75	0.84	−1.54	1.00	0.32	
VER A5	235.65	240	0.57	1.05	0.55	1.04	0.53	0.58	0.56	
VER A6	241.81	244	0.53	1.06	0.59	1.07	0.86	−0.18	0.86	

VER B1	228.32	243	0.74	1.01	0.10	0.98	−0.27	0.40	0.69	0.71
VER B2	256.85	243	0.26	1.12	1.20	0.99	−0.13	1.63	0.10	
**VER B3**	195.49	244	0.99	0.87	−0.97	0.97	−0.31	-4.14	**0.00**	
**VER B4**	155.09	242	1.00	0.71	−1.60	0.89	−0.81	−5.20	**0.00**	
VER B5	251.09	243	0.35	1.10	1.12	1.07	0.98	1.46	0.15	
VER B6	255.70	244	0.29	1.12	0.92	1.02	0.25	1.66	0.10	

DES A1	229.14	242	0.71	1.06	0.42	1.04	0.44	1.13	0.26	0.70
DES A2	202.31	243	0.97	0.94	−0.26	0.95	−0.38	0.86	0.39	
DES A3	221.41	243	0.84	1.02	0.20	1.07	0.70	1.53	0.13	
**DES A4**	269.71	241	0.10	1.23	**2.09**	1.15	1.81	−3.32	**0.00**	
DES A5	161.91	243	1.00	0.78	−0.81	0.94	−0.37	−1.05	0.29	
DES A6	232.11	242	0.66	1.07	0.72	1.02	0.26	−0.03	0.98	

DES B1	186.90	244	1.00	0.88	−0.69	0.95	−0.41	−0.22	0.83	0.68
DES B2	227.82	243	0.75	1.05	0.32	0.95	−0.46	1.05	0.30	
DES B3	198.46	242	0.98	0.93	-0.36	1.00	0.02	0.87	0.38	
**DES B4**	330.31	242	0.00	1.47	3.51	1.18	**2.15**	−3.29	**0.00**	
DES B5	160.54	243	1.00	0.77	−0.84	0.91	−0.53	−0.28	0.78	
DES B6	167.75	242	1.00	0.80	−1.65	0.85	−1.77	1.76	0.08	

**n* = 245 (Chile: *n* = 169; Germany: *n* = 76); DES = anger-oriented strategies; KON = palliative strategies; PRO = problem solving; SOZ = seeking social support; VER = avoidant coping; Situation A = social situation (arguing with a friend); Situation B = academic situation (problems with homework); *p*_χ__2_ = *p*-value for pearson χ^2^ test; *OUTFIT*_*MSQ*_ = outfit-mean-square statistic; *INFIT*_*MSQ*_ = infit-mean-square statistic; *OUTFIT*_*zSTD*_ = *Z*-standardized outfit statistic; *INFIT*_*zSTD*_ = *Z*-standardized infit statistic, values above 1.964 or below −1.964 in bold face; concurent calibration approach for itemparameter estimation based on the total sample (Germany and Chile); Splitting by country for Fischer-Scheiblechner Test; *r*_*WLE*_ = reliabilities of the weighted likelihood estimates.*

In the following the contents of the items with DIF are given. Of the scale *anger-oriented strategies* (DES) the item *“I totally freak out”* showed DIF in both situations (DES, A4: *Z* = −3.32; *p* = 0.001; Des, B4: *Z* = −3.29; *p* = 0.001). Chilean children reported to show this kind of reaction more frequently than German children. Regarding *palliative strategies* (KON), DIF was shown for the item *“I allow myself to take a break”* (A3: *Z* = 5.02; *p* = 0.000; B3: *Z* = 2.82; *p* = 0.005). German children reported to react in this way more frequently in both situations than Chilean children. Further, Chilean children reported more often *“I do something that I really enjoy”* than German children in the social situation (A6: *Z* = −2.16; *p* = 0.031).

Moreover, Chilean children agreed with the statements (Items) “*I make myself comfortable”* (German: “*dann mache ich es mir erst mal richtig bequem”;* Spanish: *“me pongo en un lugar comodo”;* KON, B4: *Z* = −2.42; *p* = 0.016) and *“I take a rest”* (B1: *Z* = −2.38; *p* = 0.017) more frequently in the academic situation than German children.

Of the *avoidant coping* (VER) scale, Chilean children agreed with the item “*I tell myself everything will work out on its own”* (VER, B3: *Z* = −4.14; *p* = 0.000) as well as with the item *“I act like everything is alright”* (VER, B4: *Z* = −5.20; *p* = 0.000) more frequently in the academic situation than German children.

Regarding the scale *seeking social support* (SOZ), DIF was shown for the item “I tell someone how I felt” (SOZ, B4: *Z* = −2.68; *p* = 0.007). Chilean children agreed with this statement more frequently in the academic situation than German children.

## Discussion

In the present research, we analyzed the appropriateness of a translation and cultural adaption approach to assess emotion regulation strategies in Chile. Overall, the results indicate that the Spanish language adaptation of the German SSKJ 3-8 (Lohaus et al., 2006), which was adapted to the Chilean cultural context, is psychometrically comparable to the German source version. The results indicate that the five scales across the two situations of SSKJ 3-8 show satisfactory reliabilities based on the total sample (Germany and Chile). As hypothesized, the scaling model divided by the two situations (i.e., social and academic situation) fits better than a scaling model across the two situations and across the cultural contexts (i.e., Chilean and German subsamples). This finding is in line with past research, which proposed to assess emotion regulation strategies separately for specific situations (Causey and Dubow, 1992; Lohaus et al., 2006). In contrast to the test manual of the SSKJ 3-8 (Lohaus et al., 2006), which proposes that a summative evaluation across the two situations is also possible, based on our results we strongly recommend to assess emotion regulation strategies separately for the social and the academic situation. It turned out that only for the scale SOZ the differentiation between the two situations has less influence on the one-dimensional model fit. This result could indicate that the willingness to look for social support might be a more global strategy, which is less situation specific than other emotion regulation strategies.

Regarding the cultural context, the results show that the Spanish language adaptation of the SSKJ 3-8 is psychometrically comparable to the German version with only few items showing minor deviations. For instance, there was a difference between the German and the Chilean subsamples in the item *“I totally freak out”* (German: “*dann raste ich total aus”;* Spanish: *“me enfurezco*”) of the scale *DES*. Chilean children reported to use this strategy more frequently than German children did. Former research has shown that DES differ among cultural contexts (Cole et al., 2006). In interdependent contexts – as well as based on general Latin-American values (*simpatía*, *familismo*, *respeto*) – interpersonal harmony is encouraged and negative emotional expressions like anger are avoided. In contrast, in independent contexts the expression of anger might be instrumental to achieve individual goals (Triandis et al., 1984; Halgunseth et al., 2006; Trommsdorff, 2009). In general, in independent contexts, for instance in Germany, children are encouraged by parents to express emotions as dissatisfaction or anger in order to contribute to children’s development of self-assertiveness. In interdependent contexts, parents undermine reactions of negative emotions by downplaying the frustration event and helping to accept the situation (Trommsdorff, 2009; Jaramillo et al., 2017). Thus, one would expect that German children report to use DES more often than Chilean children did. In fact, analyses of cultural differences with these German and Chilean subsamples support this (Weis et al., 2016). We suppose that the difference in the specific item “*I totally freak out”* might be rooted in translation issues or cultural language differences. The Spanish translation *“me enfurezco*” might be weaker and less negative than the German: “*dann raste ich total aus*.”

Similarly, the difference which we found between the German and the Chilean subsamples for the item “I allow myself to take a break” (German: “*dann gönne ich mir erst mal eine Pause*”; Spanish: “*hago una pausa*”) might be caused by language differences. This item has been changed after the pilot study for reasons of comprehensibility and these might have appeared due to cultural language differences. The German expression might be cultural specific and has a rather positive connotation. This might be a reason for the translation difficulties as well as a reason why German children reported to use this strategy more frequently than Chilean children did.

In contrast, the result that Chilean children reported to use the strategy “I tell someone how I felt” (German: *“dann erzähle ich jemandem, wie ich mich dabei gefühlt habe”*; Spanish: *“le cuento a alguien como me sentí en esa situación”*) more frequently than German children, might be explained by differences in cultural values. The higher importance of interdependent values as well as the Latin-American values *simpatía* and *familismo* in Chile might make it more cultural appropriate to tell others about the own feelings.

Following the strategies on achieving measurement invariance in large-scale-assessments [OECD, 2014, p.148 (report on “dodgy” items)], a pragmatic solution would be to simply delete items, which show DIF, from scaling. However, this might shorten the scales and a more sophisticated method of item translation or formulation might enhance the specific item equivalence. This could be an issue for further research.

Culture specific socialization practices might lead to differences between countries in emotion regulation strategies (Trommsdorff, 2009; Jaramillo et al., 2017) as well as in their relations with other constructs like emotional problems (i.e., anxiety, aggression etc.) or academic achievement. Thus, another interesting question for future research could be to compare relations between the emotion regulation strategies of the SSKJ 3-8 with other variables across countries. Moreover, possible moderator variables such as gender (Eschenbeck et al., 2007; Weis et al., 2013), parents’ level of education, and the socioeconomic status of the family (e.g., Kağitçibasi, 1996; Sektnan et al., 2010; Størksen et al., 2014) could be included in further studies with larger samples.

### Strengths and Limitations

The present study shows that a careful translation and cultural adaptation process including discussions with native speakers (in both languages) has a positive impact on the psychometric properties of the resulting translated instrument. A possible drawback of the current paper are the rather small sample sizes, giving just a first impression of general measurement invariance between the German and Chilean version of the SSKJ 3-8. However, a strength of this study is that we were able to implement an IRT-analysis approach. We used the package *pairwise* (Heine, 2019), which offers a non-iterative method for item parameter determination named *PAIR* and enables stable item parameter estimates of rather small sample sizes (Heine and Tarnai, 2015; Heine et al., 2018). A further merit of the study is the comparison between the two scaling models. Even though we could not conduct the comparison separately for both subsamples due to rather small sample sizes, the results underline the importance of the distinction between the two situations in the SSKJ 3-8. Future research should investigate similarities and differences between subsamples regarding different scaling models of the SSKJ 3-8.

## Conclusion

This study showed that the Spanish language adaptation of the German Stress and Coping Questionnaire for Children and Adolescents (SSKJ 3-8) is comparable to the German version and is a reliable instrument to assess emotion regulation strategies in Chile. Thus, this Spanish language adaptation might be useful for diagnostic purposes of practitioners as well as for research in Chile.

## Data Availability Statement

The raw data supporting the conclusions of this article will be made available by the authors to any qualified researcher after personal contact.

## Ethics Statement

All procedures performed in studies involving human participants were in accordance with the ethical standards of the institutional research committee and with the 1964 Helsinki Declaration and its later amendments or comparable ethical standards. For all minors who participated in the study, the parents signed a written declaration of consent. All parents were informed about the study with an information sheet and gave their informed consent.

## Author Contributions

MW was responsible for the conception and design of the study as well as for the data collection in Chile. J-HH was responsible for the data analysis and interpretation. MW and J-HH wrote, revised, and approved the manuscript.

## Conflict of Interest

The authors declare that the research was conducted in the absence of any commercial or financial relationships that could be construed as a potential conflict of interest.

## Footnotes

1 For the English wording of the scales and items of the SSKJ 3-8 by Lohaus et al. (2006) we refer to Eschenbeck et al. (2012) throughout the whole manuscript. For the German wording of the scales and items of the SSKJ 3-8 by Lohaus et al. (2006) we refer to Eschenbeck et al. (2006) throughout the whole manuscript. Source of the Spanish version of the questionnaire is Lohaus et al. (2006) by Hogrefe Verlag GmbH & Co., KG, Göttingen.

2 The original German item “dann fluche ich vor mich hin” (“I curse to myself”) was translated into “maldigo” in the first Spanish version. As the pilot study showed that this item was difficult to understand for the target population, it was changed to “digo garabatos en voz baja.” Similarly, the item “dann gönne ich mir erst mal eine Pause” (“I allow myself to take a break”) was first translated into “me permito hacer una pausa” and was changed to “hago una pausa” after the pilot study.
